# Advancing Cheminformatics—A Theme Issue in Honor of Professor Jürgen Bajorath

**DOI:** 10.3390/molecules27082542

**Published:** 2022-04-14

**Authors:** Martin Vogt

**Affiliations:** Department of Life Science Informatics, B-IT, LIMES Program Unit Chemical Biology and Medicinal Chemistry, Rheinische Friedrich-Wilhelms-Universität, Friedrich-Hirzebruch-Allee 5-6, 53115 Bonn, Germany; martin.vogt@bit.uni-bonn.de

While cheminformatics problems have been actively researched since the early 1960s, as witnessed by the QSAR approaches developed by Toshio Fujita and Corwin Hansch [1], its growth into an independent research field might be signaled by the definition given by Frank K. Brown in 1998, stating that

“*Chem(o)informatics is the mixing of those information resources to transform data into information and information into knowledge for the intended purpose of making better decisions faster in the area of drug lead identification and optimization*”.

[2]

Taking this year as the, admittedly completely arbitrary, “birth year” of cheminformatics as an independent field, it coincides closely with Prof. Jürgen Bajorath’s research path, publishing his first purely chemoinformatic papers in 1999 [3,4]. Rightly so, Prof. Bajorath belongs to the first generation of “full time professional” cheminformaticians, and he has significantly influenced and advanced the field since then for more than twenty years. He received his PhD in structural biochemistry in 1988 from the Freie Universität Berlin, and his initial postdoctoral research focused on molecular modeling and drug design. Around the turn of the millennium, during his stay at New Chemical Entities–AMRI Bothell Research Center as director for computer-aided drug discovery and as an affiliate professor at the University of Washington, his research interests shifted to the study of more general methodological aspects of drug design, initially focusing on the study of effective molecular descriptors and short fingerprint representations for molecule classification [3,4].

In 2004, he became a full-time professor at the University of Bonn where he holds the chair of life science informatics. Here, he has established the international life science informatics master program, uniquely combining bio- and chem-informatics topics into a single curriculum aimed to attract students from all over the world. In the 17 years since, his research has covered an extraordinarily wide range of topics from molecular modeling, similarity searching, and data visualization to statistical data analysis, machine learning and deep learning, as witnessed by well over 500 publications from 2006 to 2021 alone. During this period of his tenure at the University of Bonn, Prof. Bajorath has been highly successful in nurturing the next generation of cheminformaticians. He has supervised more than 30 PhD candidates from different backgrounds ranging from biology, chemistry and pharmacology to bioinformatics and computer science. Their varied research and scientific contributions can be seen as representative of the diverse and evolving field of cheminformatics as a whole and have been vital in establishing and/or promoting key concepts such as “activity landscape” [5], “activity cliff” [6], “chemical space network” [7], “SAR index” [8], “SAR transfer” [9], and “SAR matrix” [10], which are now strongly associated with Prof. Bajorath’s work.

His contributions have been well-recognized by the scientific community, and in 2015 he received the prestigious Herman-Skolnik Award and in 2018 the National Award for Computers in Chemical and Pharmaceutical Research from the American Chemical Society. Furthermore, in 2016, he received the inaugural Fujita Award of the Hansch–Fujita Foundation.

If one were to define the broad research of Prof. Bajorath in a single phrase, it might be “method development for qualitative and quantitative structure activity/property relationships”. Of course, structure activity relationship (SAR) and structure property relationship (SPR) research themselves are at the heart of cheminformatics and quantitative SAR studies, i.e., the mathematical modeling of SARs for prediction of activity of (analog) compounds is one of the originating research areas of cheminformatics itself [1]. However, the emphasis of his research lies not necessarily in its direct and immediate applicability but instead in its focus on gaining knowledge and understanding the relation between molecular structure and biologically relevant properties such as target-specific activity. With this focus, the utility of a mathematical model, a machine-learning classifier or a regression model does not primarily lie in its—hopefully and necessarily—reasonable accuracy and predictive power, but instead in the insights that can be gained from it regarding the mechanisms and structural features that modulate relevant biological properties.

Thus, it comes as no surprise that, while quite a few of his publications have centered on the quantitative performance of classification methods and regression models, a major part of his research focuses on method development centered on qualitative aspects of SARs. While traditional statistical and data analytical methods as well as machine-learning algorithms—in particular, in recent years, deep-learning approaches—belong to the staples of his research, the application, development, and advancement of these methods in Prof. Bajorath’s research is driven not primarily by the urge to outperform competing methods quantitatively but instead to develop approaches and methods that facilitate novel ways to gain insights and knowledge about all aspects of SARs. In this sense, quantitative performance becomes subordinate to the knowledge that can be gained from such methods and models. This aspect can be nicely illustrated using the example of his recent work on deep learning [11]. The recent advances in hardware and methodology have made the application of deep learning using deep neural networks (DNNs) almost ubiquitous in many areas, and cheminformatics is no exception [12]. These new methods have proven in many instances to be competitive or superior. However, the initial black box character of DNNs, which, by design, precludes any direct model explanation for the decisions made by DNNs, has also expedited the development of explainable artificial intelligence (XAI). From an epistemological point of view, this can be considered an even more significant development, as XAI aims to elucidate the reasons and rationales on which decisions of traditional machine-learning and deep-learning models are based. For instance, the extraction of decision-relevant specific (structural) features for compound activity or inactivity can lead to a better understanding of SAR relationships [11]. In this context, the application of machine-learning models for activity prediction becomes a diagnostic tool that helps one better understand the underlying mechanics of the relationship between molecules and their activities and properties in a biological context [11].

The activity landscape concept [5] might be the most representative and illustrative example of Prof. Bajorath’s research integrating qualitative and quantitative aspects of SARs. It extends the idea of chemical space where molecules are organized in high- (or low-) dimensional metric spaces, where distance between molecules relates to (structural) similarity, however it may be defined. The attribution of biological activities or properties then leads to activity or property landscapes. These concepts are, by their inherent nature, highly abstract, and the concrete realization of activity landscapes can take very different forms that lead to qualitative and/or quantitative SAR descriptions. This topological analogy of SARs has proven to be very successful in integrating observed SAR phenomena in a common framework. The structure property principle, stating that similar molecules have similar biological properties, and, on the opposing end, the observation that some highly similar molecules can still have very different activities with respect to the same target, translate into the geographical features of gently rolling hills and sharp cliffs, which are now known as “activity cliffs” in the cheminformatics world [6]. The different ways in which activity landscapes can be realized and visualized are highlighted in Prof. Bajorath’s work, ranging from topographical 3D maps [13] and network representations such as network-like similarity graphs [13] or chemical space networks [7], to multi-target activity landscapes [14] and image analysis based on 2D and 3D (heat-)maps [15].

This theme issue in honor of Prof. Bajorath comprises a collection of original wyresearch articles and reviews by colleagues, collaborators, former PhD students, and fellow researchers from the field of cheminformatics, and showcases the spectrum of the field, which has been significantly shaped by Prof. Bajorath’s research.
